# Novel reusable hydrogel adsorbents for precious metal recycle

**DOI:** 10.1038/s41598-021-99021-5

**Published:** 2021-10-01

**Authors:** Thakshila N. Dharmapriya, Ding-Yang Lee, Po-Jung Huang

**Affiliations:** Institute of Environmental Engineering, National Sun Yat-Sen University, Kaohsiung, 80432 Taiwan

**Keywords:** Gels and hydrogels, Organometallic chemistry

## Abstract

A novel polyethylene glycol diacrylate-allylthiourea (ATU-PEGDA) hydrogel was simply synthesized through photo-reaction. Modified thiourea simultaneously employed chelation and electrostatic force to selectively recycle Ag(I) and Pd(II) from electrolytic wastewater. Sorption efficiency was nearly 100% for Ag(I) and Pd(II), which occurred at initial pH of 1 within 300 min. The adsorption characteristics of ATU-PEGDA followed Langmuir isotherm model and the maximum adsorption capacity of Ag(I) and Pd(II) achieved 83.33 and 152.81 mg g^−1^ sorbent, respectively where Pseudo-first order model demonstrate the adsorption kinetics. In the presence of other heavy metals, ATU-PEGDA performed high selectivity, 0.89 and 1.31 towards Ag(I) and Pd(II). ATU-PEGDA can be completely regenerated within 120 min using 0.5 M thiourea—0.001 M HNO_3_ and 1 M thiourea—4 M HCl after the adsorption of Ag(I) and Pd(II), respectively. Thiourea-branched structure was created after regeneration, improving the adsorption capacity. Compared to initial hydrogel, the adsorption capacity of Ag(I) and Pd(II) increased 31.83 ± 3.08% and 75.12 ± 11.02%, respectively. Over 10 consecutive adsorption–desorption cycles, ATU-PEGDA performed 111.34 and 263.79 mg g^−1^ sorbent in adsorption capacity of Ag(I) and Pd(II). Chromism of ATU-PEGDA hydrogel was a benefit to determine adsorption saturation and completely desorption of Ag(I) and Pd(II). Potentially, ATU-PEGDA can be extended to industrial applications.

## Introduction

Among various heavy metals, silver and palladium are extensively used and have special economic interest than many other metals in various industrial aspects such as electronics, jewelry and ornaments, chemical engineering and medication, etc.^1–6^. However, this has brought potential hazards to the environment specially for the aquatic systems. Wastewater containing these precious metals cause an adverse effect on human and animal bodies due to their toxicity. Consequently, it is necessary to develop effective methods to remove and recover these metals from wastewater^7–10^. Traditional methods of remove and recover of these metals are solvent extraction^11^, ion-exchange^12,13^, membrane separation^14^, electrolysis^15^ precipitation^16^ ion flotation^17^ reverse osmosis^18^ and adsorption^19^ etc. Among them, adsorption is an emerging method where an adsorbent is used to adsorb metal ions onto the adsorbent by ion exchange, electrostatic interaction, physical adsorption, or coordination interactions^20^. Adsorption is considered to be one of the most efficacious way since adsorbent materials can be easily synthesized with high efficiency, selective for specific metal ions and in economical point of view, most of them are cheap and environment friendly^1,5,9^. Variety of adsorbents have been developed and tested to adsorb metal ions form waste water including activated carbon^21^, biomass adsorbents^22^, inorganic minerals^23^ and chelating resins^7^ etc. Among them, chelating resins are intensively focused due to their efficient adsorption capacity and high selectivity towards metal ions. A chelating resin essentially consists of two components as the functional group which form the chelate interaction with ions and the polymeric matrix which acts as the support. The functional groups in the polymers contain one or two donor atoms that protonate in a low pH medium resulting in complex formation with metal ions^5,9^. They often contain amino, thio, carboxy, oxo, or phosphoryl as chelating ligands where N, S, O, and P acting as donor atoms^1,5,7^. According to the Hard–Soft Acid–Base (HSAB) theory by Pearson; soft metal ions, for instance; Ag(I), Au(III), and Pd(II) ions show affinity towards soft bases with donor atoms N and S^3,5,7^.

Poly(ethylene glycol) Diacrylate (PEGDA) is an extremely hydrophilic, having three-dimensional network structure and colorless hydrogel. This is a polymer that is formed through certain chemical and physical cross-linking while density is less than that of traditional resins. As the water content increases, the volume is expanded and the pore size is increased of it. Because of its high hydrophilicity, non-toxicity, photodegradable, and biocompatibility, it is widely used in chemical, cosmetic products and medicine including stem cell transplantation, contact lenses, etc.^24^.

In this work, allylthiourea (ATU) modified PEGDA hydrogel was synthesized through a simple one-step polymerization reaction under UV radiation. ATU has functional groups with chelating properties for precious metal ions. This was used as an adsorbent to remove and recover Ag(I) and Pd(II) from electrolytic wastewater. Effect of pH value, contact time, selectivity, desorption time and regeneration, competitive ions, and finally application for on-site waste electrolyte on Ag(I) and Pb(II) adsorption were discussed. In addition; isotherms and kinetics were used to fit the experimental data. It was found that the hydrogel had efficient, faster adsorption capacity. After desorption and regeneration, the adsorption efficiency for Ag(I) and Pd(II) was improved while maintaining more than 10 times reuse proving high application value.

## Results and discussion

### Characterization of ATU-PEGDA adsorbent

**Figure 1:**
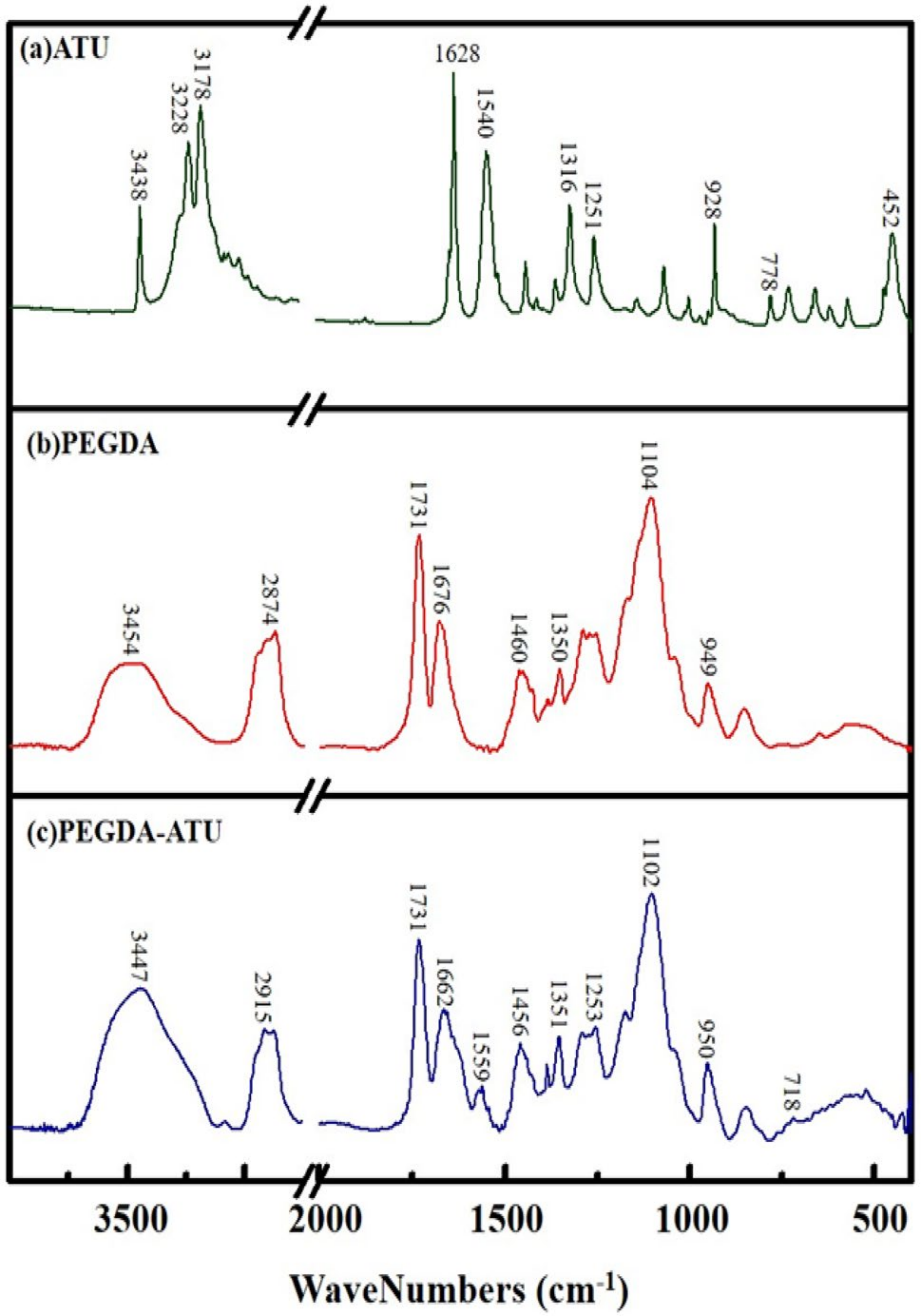
FT-IR spectra of (**a**) ATU; (**b**) PEGDA; and (**c**) PEGDA-ATU.

The FT-IR spectra of PEGDA hydrogel, ATU, and ATU-PEGDA modified hydrogel are shown in Fig. 1. Figure 1a shows the FT-IR spectrum of ATU. The adsorption bands at 3438, 3228 and 3178 cm^−1^ are assigned to –NH_2_ asymmetric stretching vibration, –NH_2_ Symmetric stretching vibration and deformation of ATU respectively. Bands 1628 and 1316 cm^−1^ are assigned to –NH_2_ deformation, 1540 cm^−1^, 1251 cm^−1^, 928 cm^−1^, 778 cm^−1^, 452 cm^−1^ are assigned to HC–N stretching and bending, CN Stretching vibration, =CH_2_ wag vibration, C=S vibration and N–CH deformation with –NH_2_ twist, respectively. Figure 1b shows the FT-IR spectra of PEGDA hydrogel. It can be observed that the adsorption band at 3454 cm^−1^ is due to –OH which is caused by H_2_O in the air. It is due to the high hydrophilicity of the hydrogel. Adsorption bands at 2874 cm^−1^ and 1460 cm^−1^ are revealing the Stretching vibration and bending vibration of –CH. The band around 1731 cm^−1^ is attributed the tensile vibration of main characteristic functional group of PEGDA hydrogels and that is ester O=CO. Adsorption bands at 1676 and 949 cm^−1^ confirm the C=C bending vibration and 1350 cm^−1^, 1104 cm^−1^ are attributed vibration of alcohol –OH bending and tensile vibration of COC respectively. The FT-IR spectra of ATU-PEGDA modified hydrogel is shown in Fig. 1c. The adsorption band at 3447 cm^−1^ is caused by both water vapor in the air and the asymmetric and symmetric stretching vibrations of ATU. Tensile vibration of ester O=CO and COC at 1731 and 1102 cm^−1^ respectively are the characteristic peaks of PEGDA, while the characteristic signal of ATU is occurred at 1559 cm^−1^ that is due to the HC–N stretching and bending deviation of 1540 cm^−1^. According to the above analysis results, the characteristic signals of PEGDA and ATU can be found on the FT-IR spectrum of ATU-PEGDA modified hydrogel and confirm that hydrogel was successfully modified and synthesized.

### Effect of pH on adsorption capacity

**Figure 2:**
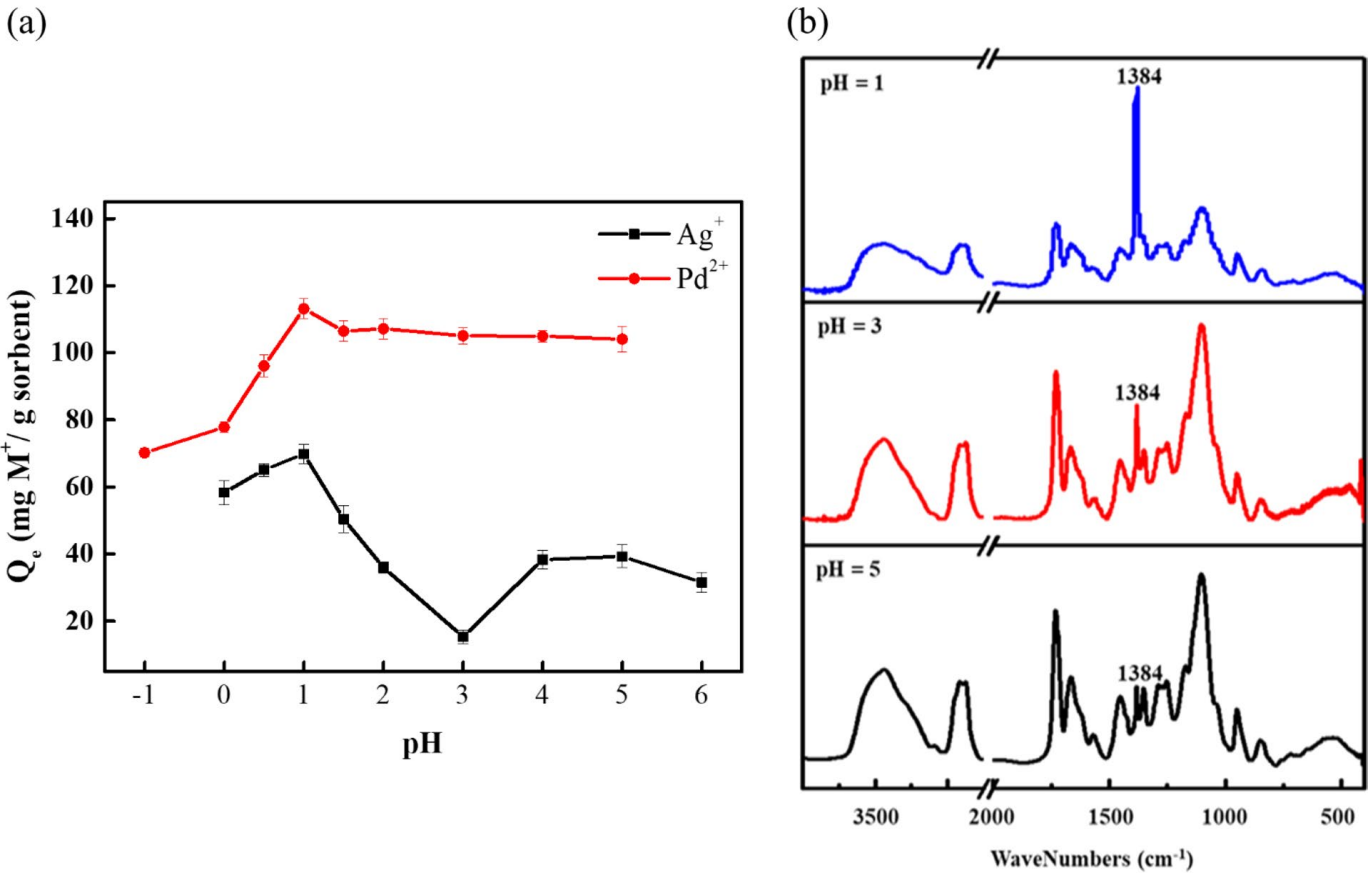
Effect of pH on adsorption capacity. (**a**) Adsorption capacity of ATU-PEGDA under different pH of the solution toward Ag(I) and Pd(II). (**b**) Differences in FT-IR spectra of PEGDA-ATU hydrogel after Ag^+^ adsorption at different pH values.

The removal efficiency of precious metal ions using an adsorbent is depended on the pH value of the solution medium^20^. Figure 2a presented the effect of different pH of the solution on adsorption of Ag(I) and Pd(II). The results demonstrated that the maximum adsorption capacity of Ag(I) ions occurred at pH 1 and it was 69.83 mg g^−1^. As the pH value of the solution increases, the adsorption capacity of Ag(I) ions were decreased till pH 3 and then slowly rises with the increased pH value showing a V shape trending. This reaction mechanism can be described as follow^4,5,7^.

Chelation reactions:

1 $${\left({\mathrm{R}}_{1}{\mathrm{R}}_{2}{\mathrm{NH}}_{2}^{+}\right)}_{(\mathrm{sorb})}+{\mathrm{Ag}}_{(\mathrm{aq})}^{+}=\left({\mathrm{R}}_{1}{\mathrm{R}}_{2}\right)\mathrm{HN}\dots {\mathrm{Ag}}_{\left(\mathrm{s}\right)}^{+}+{\mathrm{H}}^{+}$$

2 $$\left({\mathrm{R}}_{1}{\mathrm{R}}_{2}\right)\mathrm{C}={\mathrm{S}}_{(\mathrm{sorb})}+{\mathrm{Ag}}_{(\mathrm{aq})}^{+}=\left({\mathrm{R}}_{1}{\mathrm{R}}_{2}\right)\mathrm{C}=\mathrm{S}\dots {\mathrm{Ag}}_{\left(\mathrm{s}\right)}^{+}$$

Ionic interaction:

3 $$\left({\mathrm{R}}_{3}\right){\mathrm{NH}}_{2}^{+}{\mathrm{NO}}_{3\left(\mathrm{sorb}\right)}^{-}+{\mathrm{Ag}\left({\mathrm{NO}}_{3}\right)}_{2(\mathrm{aq})}^{-}=\left({\mathrm{R}}_{3}\right){\mathrm{NH}}_{2}^{+}\mathrm{Ag}{\left({\mathrm{NO}}_{3}\right)}_{2(\mathrm{S})}^{-}+{\mathrm{NO}}_{3\left(\mathrm{aq}\right)}^{-}$$

The sulfur and amine group has a chelating effect on Ag(I) as shown in Eqs. (1) and (2). At lower pH, like pH 1, amine group in thiourea would be protonated resulting in performing positive charge as shown in Eq. (3). To consider the silver ion at pH 1, excessive nitrate would partially conjugate with Ag(I) and form silver anionic complex, Ag(NO_3_)_2_^−^. As a result, the electro-attractive force is generated between thiourea and silver ion leading to higher adsorption capacity. The adsorption capacity of Ag(I) started decreasing as increasing the pH of solution. It indicated that the concentration of existing silver anionic complex also start decreasing because of lower amount of nitrate in medium. Therefore, the electro-repulsive force is generated between protonated thiourea and Ag(I) resulting in decreasing adsorption capacity. Once the pH of medium achieve weak acidity, chelation interaction would be mainly contribution of Ag(I) adsorption^25^. This can be verified from the FT-IR spectrum (Fig. 2b) as the intensity of the adsorption band at 1384 cm^−1^ which was assigned to NO_3_^−^ was decreased with the increased pH values. The growth and decline between the formation of Ag(I)/silver anionic complex and protonation/deprotonation of thiourea mainly determine the adsorption behavior at different pH of medium. Electro-attractive force play dominantly role to Ag(I) recovery using ATU-PEGDA compared with chelation. The experimental result shows the electro-attractive force and chelation both achieve minimum at pH 3. Also, since sulfur group does not have the protonation effect, the adsorption reaction can be carried out in a stable manner under different acid–base conditions (Eq. 2)^5,7,8,26^.

In the same conditions, ATU-PEGDA had a high adsorption capacity for Pd(II) when pH 1 and the adsorption capacity remained relatively constant at pH 1.5–5. However, unlike Ag^+^; the Pd^2+^ exists in the form of chloride anions (PdCl_4_)^2−^ in the solution, and this adsorption mechanism can be described as follow^5,27,28^.

Chelation reactions:

4 $$\left({\mathrm{R}}_{1}{\mathrm{R}}_{2}\right){\mathrm{NH}}^{2+}{\mathrm{Cl}}_{\left(\mathrm{sorb}\right)}^{-}+{\left({\mathrm{PdCl}}_{4}\right)}_{\left(\mathrm{aq}\right)}^{2-}=\left({\mathrm{R}}_{1}{\mathrm{R}}_{2}\right)\mathrm{HN}\dots {\left({\mathrm{PdCl}}_{3}\right)}_{\left(\mathrm{s}\right)}^{-}+{\mathrm{H}}_{\left(\mathrm{aq}\right)}^{+}+{2\mathrm{Cl}}_{\left(\mathrm{aq}\right) }^{2-}$$

5 $$\left({\mathrm{R}}_{1}{\mathrm{R}}_{2}\right){\mathrm{C}=\mathrm{S}}_{\left(\mathrm{sorb}\right)}+{\left({\mathrm{PdCl}}_{4}\right)}_{(\mathrm{aq})}^{2-}=\left({\mathrm{R}}_{1}{\mathrm{R}}_{2}\right)\mathrm{C}=\mathrm{S}\dots {\left({\mathrm{PdCl}}_{3}\right)}_{\left(\mathrm{s}\right)}^{-}+{\mathrm{Cl}}_{\left(\mathrm{aq}\right)}^{-}$$

Ionic reaction:

6 $$\left({\mathrm{R}}_{1}{\mathrm{R}}_{2}\right){\mathrm{NH}}_{(\mathrm{sorb})}+{\mathrm{HCl}}_{(\mathrm{aq})}=\left({\mathrm{R}}_{1}{\mathrm{R}}_{2}\right){\mathrm{NH}}_{2(\mathrm{sorb})}^{+}+{\mathrm{Cl}}^{-}$$

7 $$\left({\mathrm{R}}_{1}{\mathrm{R}}_{2}\right){\mathrm{NH}}^{2+}{\mathrm{Cl}}_{(\mathrm{sorb})}^{-}+{\left({\mathrm{PdCl}}_{4}\right)}_{\left(\mathrm{aq}\right)}^{2-}=\left({\mathrm{R}}_{1}{\mathrm{R}}_{2}\right)\mathrm{HN}\dots {\left({\mathrm{PdCl}}_{4}\right)}_{\left(\mathrm{s}\right)}^{-}+{\mathrm{Cl}}_{\left(\mathrm{aq}\right)}^{-}$$

Pd(II) adsorption on the ATU-PEGDA may be due to ionic interaction between protonated amine groups and chloro-palladate complexes or chelation of Pd(II) and N or S donor atoms on the adsorbent or both mechanisms. The possible reactions are given by Eqs. (4)–(7). At low pH values, in other words in high HCl concentrations, the excess of chloride induces a strong competition effect that limits the adsorption effect of PdCl_4_^2−^^5^.

### Adsorption kinetics and isotherm

**Figure 3:**
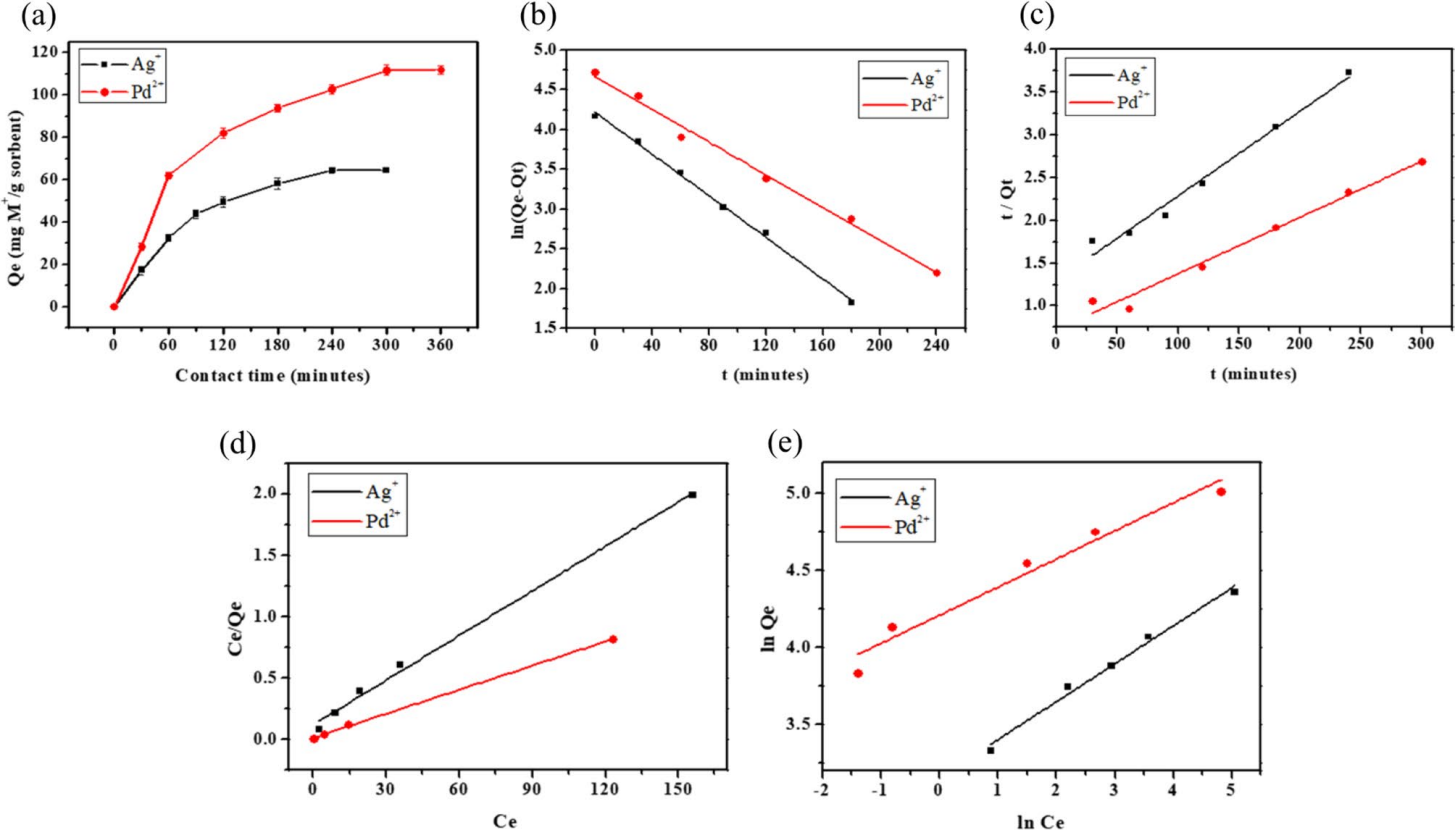
The adsorption behavior of Ag(I) and Pd(II) using ATU-PEGDA. (**a**) Contact time; (**b**) fitting results of pseudo-first-order kinetic model; (**c**) fitting results of pseudo-second-order kinetic model; (**d**) fitting results for the Langmuir; (**e**) fitting results fro the Freundlich isotherm.

All adsorption experiments for Ag(I) and Pd(II) using ATU-PEGDA occurs under their optimized conditions. Figure 3a presents contact time of Ag(I) and Pd(II) and ATU-PEGDA reached its equilibrium stage after 240 and 300 min, respectively. Pseudo-first-order and pseudo-second-order model are used to describe the adsorption kinetics of Ag(I) and Pd(II). Their fitting results were presented in Fig. 3b,c. The correlation coefficient (R^2^) of pseudo-first-order kinetic model for adsorption were 0.99 for both metals and for pseudo-second order kinetic model they were 0.97 for both metals. The pseudo first-order model suggests that the adsorption mechanism is attributed to physical process representing reversible adsorption process. The pseudo-first order kinetic model is primarily affected by one reactant concentration^29^. For example, the major adsorbates are ${({\mathrm{AgNO}}_{3})}_{2}^{-}$ and NO_3_^−^ when pH is 1. The concentration of nitrate was approximately 100 times higher than silver ion. Therefore, the concentration change of nitrate is not obvious compared with silver ion. In this study, the adsorption kinetics would be described through pseudo-first order reaction. The adsorption behavior of Pd(II) using ATU-PEGDA also can be observed in its kinetic model. The excessive chloride ion existed at pH 1 during adsorption. As a result, pseudo-first order reaction would completely demonstrate the adsorption process of ATU-PEGDA toward Pd(II). Moreover, the equilibrium adsorption capacities of Ag(I) and Pb(II) ions calculated from the pseudo-first-order (Q_e_) were 67.89 and 105.99 mg g^−1^ respectively which were consistent with the experimental value 64.63 mg g^−1^ and 111.99 mg g^−1^, respectively. Furthermore, rate constants (k_1_) of Ag(I) and Pd(II) adsorption were 0.0301 and 0.0236 min^−1^ respectively, which revealed that the ATU-PEGDA could remove the metal ions from the aqueous medium in fast, and the adsorption rate followed the order Ag(I) > Pd(II)^20^. Table S1 shows the results of pseudo-first-order and pseudo-second-order models that were used to evaluate the kinetic mechanism which controls the adsorption process. Qing et al. observed that higher adsorption rate of gold (III) compared with Ag(I) and Pd(II) using TiO_2_ nanoparticles and the binding affinity plays an important role in adsorption^30^. Mohammad Ziaul Hyder et al. also synthesized thiocarbamate grafted polymer and the adsorption rate of Au(III) is the fastest because of its higher binding affinity^31^. Therefore, ATU-PEGDA might perform high binding affinity toward Ag(I) than Pd(II) resulting in higher adsorption rate of Ag(I). The effective diffusion coefficient was determined by Fick’s second law within homogeneous semi-infinite medium^32^. The formula was shown as below

$$\mathrm{C}\left(\mathrm{x},\mathrm{t}\right)={C}_{0}erfc(\frac{x}{2\sqrt{{D}_{e}t}})$$

where C(x,t), x is the distance of Ag(I) or Pd(II) travelling; t is the time in minute; C_0_ is the initial concentration in ppm; C(x,t) is the concentration of Ag(I) or Pd(II) in solution at equilibrium; D_e_ is the effective diffusion coefficient in mm^2^ min^−1^ for Ag (I) or Pd(II) in ATU-PEGDA. All parameters for determining are chosen at equilibrium.

The effective diffusion coefficient of Ag(I) and Pd(II) are 9.64 × 10^–3^ and 2.82 × 10^–3^ mm^2^ min^−1^, respectively. This result also demonstrated that the adsorption rate of Ag(I) is faster than that of Pd(II).

Figure 3d,e) describe the fitting curves of the Langmuir and the Freundlich isotherm models while table S2 represents the results of the Langmuir and the Freundlich isotherm model for Ag(I) and Pd(II) adsorptions. Langmuir isotherm model can fully describe the adsorption behavior of ATU-PEGDA toward Ag(I) and Pd(II), compared with Freundlich isotherm model. The Langmuir model describes that a monolayer adsorption process happens on a homogenous adsorbent surface and the adsorption energy of all active sites on the adsorbent is always similar^25^. It indicated that silver ion only interacted with ATU through chelation reactions resulting in monolayer adsorption. The maximum adsorption capacities from Langmuir isotherm model are 83.33 mg g^−1^ sorbent and 152.91 mg g^−1^ sorbent toward Ag(I) and Pd(II), respectively The ratio of ATU to PEGDA in this study is 6 to 1 indicating 552.97 mg of ATU would theoretically exist on PEGDA network. According to the adsorption mechanism, one thiourea would react with three Ag(I) or Pd(II) through electrostatic force and chelation reaction. Therefore, the maximum adsorption capacity might be 1540.36 mg Ag(I) and 1519.68 mg Pd(II) per gram of ATU-PEGDA adsorbent. However, the maximum adsorption capacity fitted by Langmuir model was quite lower than theoretical adsorption amount. The main reason is that the functionalization of PEGDA was processed by UV irradiation. The vinyl group in PEGDA was not only used as cross-linker but also considered as grafting site for modification. Therefore, the modifier, ATU would not completely conjugate with PEGDA resulting in obviously lower adsorption capacity compared with theoretical calculation.

**Figure 4:**
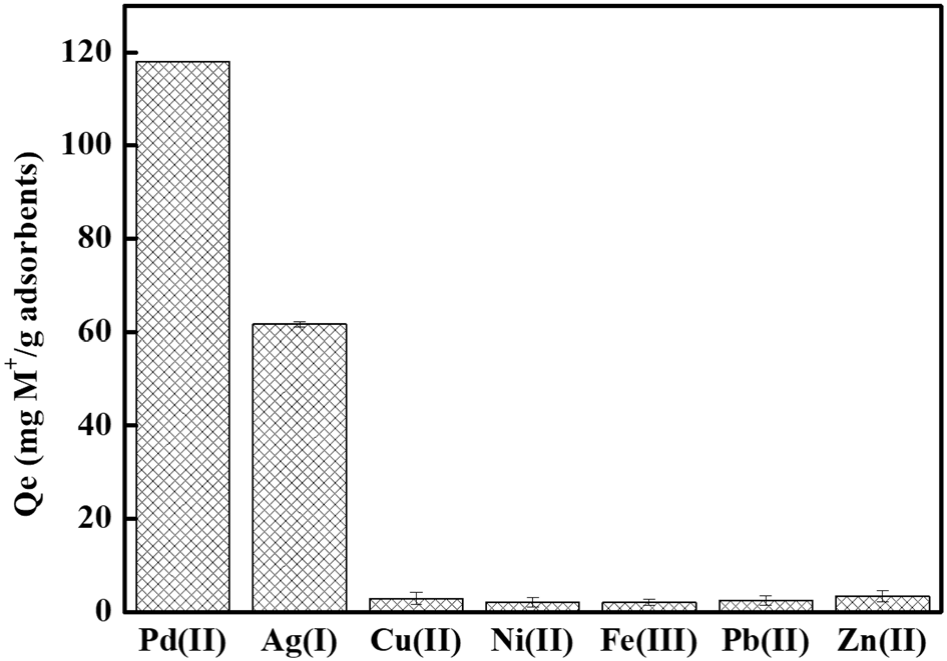
Selective adsorption of ATU-PEGDA for multiple metal ions under pH 1 condition at 25 °C. The initial concentration of each metal ion was 100 ppm.

In addition, selectivity is also a salient factor hence the selective adsorption of Ag(I) and Pd(II) with co-existing ions by ATU-PEGDA were determined separately. As shown in Fig. 4, ATU-PEGDA performed high selectivity for Ag(I) and Pd(II) and the adsorption capacities achieved 61.61 and 118.12 mg g^−1^, respectively. The difference of adsorption amount between Ag(I) and Pd(II) is mainly caused by their ionic types in solution. Silver performed two ionic types, Ag(I) and Ag(NO_3_)_2_^−^ complexes varied in different pH. While Pd(Cl_4_)^−^ is the only type in aqueous solution. At acidic environment, like pH 1, the ionic exchange ability dominantly contribute the adsorption. Therefore, repulsive force would possibly be formed in the case of Ag(I). As a result, the adsorption amount of Ag(I) was lower than that of Pd(II). Remaining heavy metals only accounted for 1.6–5.7% adsorption capacity in Ag(I)/Pd(II) containing solution which were almost negligible. Adsorption selectivity of Ag(I) and Pd(II) was calculated using experimental data (Table S3) and values were 0.89 and 1.31 respectively which were higher than other metals in the solution. This result of selectivity adsorption also reflects the Hard–Soft Acid–Base theory (HSAB). Hard base prefer to form stably ionic bonding with hard acid. On the other hand, soft acid-hard base pair or hard acid-soft base pair would form unstable complexes resulting in dissociation. The modifier, allylthiourea (ATU) contains sulfur and amine presenting as soft base, which is easily to chelate with soft acid (Ag(I) and Pd(II)). The remaining metal ions, like Ni(II), Pd(II), Zn(II), and Fe(II) are classified as hard-acid. Consequently, ATU would not be easy to chelate with these metal ions.

### Regeneration of spent ATU-PEGDA

**Figure 5:**
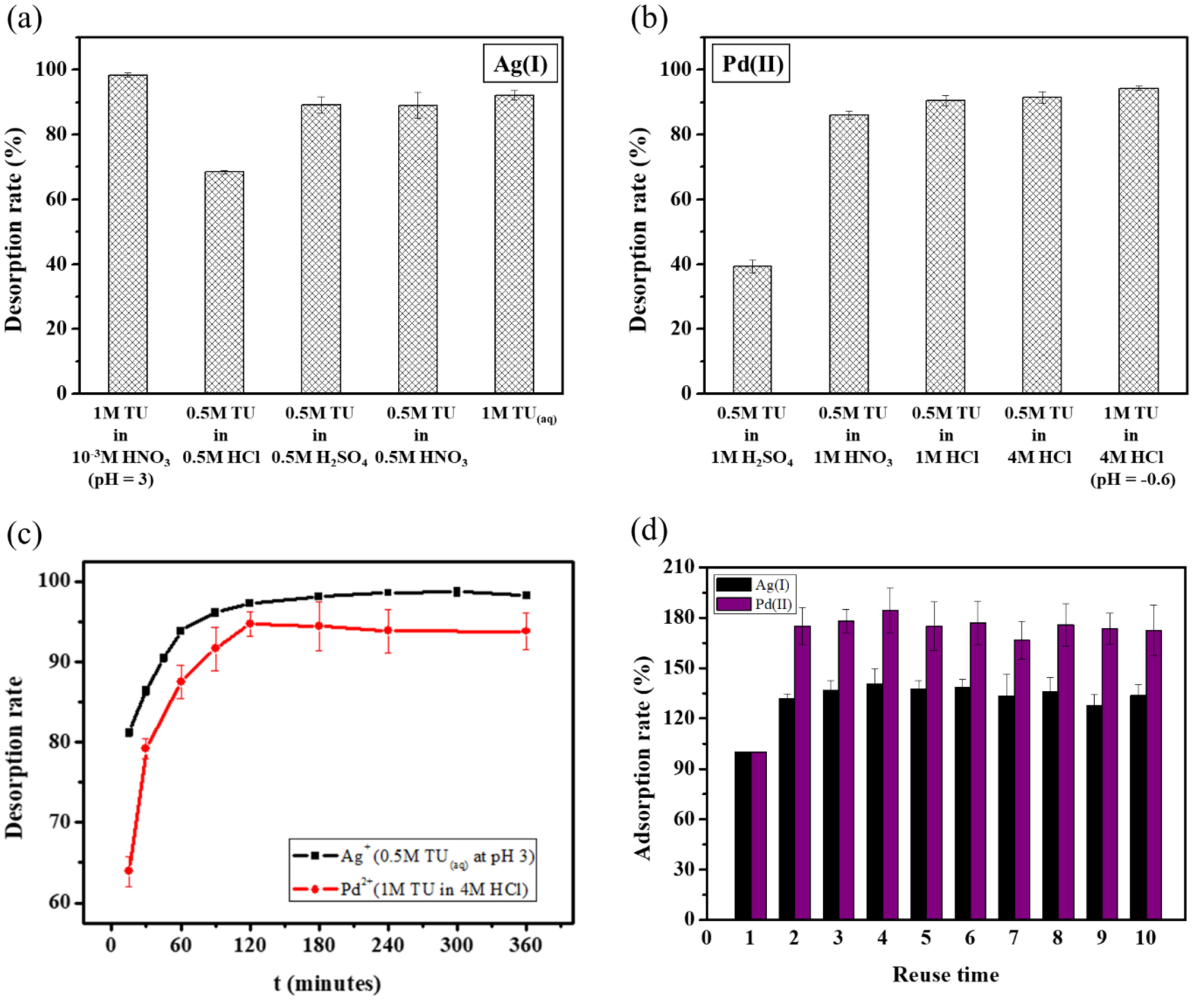
Desorption of ATU-PEGDA. (**a**) Using different eluents for silver ion; (**b**) using different eluents for palladium ion. (**c**) Desorption rate of spent ATU-PEGDA. The regeneration of silver ion and palladium ion were processed by 0.5 M TU at pH 3 and 1 M TU in 4 M HCl solution, respectively. (**d**) Adsorption–desorption cycle of ATU-PEGDA hydrogel adsorbents. (W = 5 mg, V = 10 mL, 25 °C).

To select best eluent for the desorption of Ag(I) and Pd(II), spent ATU-PEGDA adsorbents were carried out by using various concentrations of thiourea (TU) dissolved in various concentrations of HNO_3_, H_2_SO_4_ and HCl acids as desorption solutions. Figure 5a,b present the desorption rates due to the influences of different desorbing eluents on adsorbed Ag(I) and Pd(II) ions onto ATU-PEGDA adsorbents. The results show that, the desorption rate reached maximum in 1 M TU in 10^–3^ M HNO_3_ which was 98.30 ± 0.69% for Ag(I) and 1 M TU in 4 M HCl which was 94.24 ± 0.64% for Pd(II). The desorption efficiency is determined by the solubility of Ag(I) and Pd(II) in eluent. The introduction of HCl would form precipitation, like AgCl during desorption process. Therefore, the captured silver ion in ATU-PEGDA would not be easily released. Compared with Pd(II), Pd(II) easily chelate with chloride ion and nitrate ion resulting in higher desorption rate and efficiency. The pH of the eluent was set as pH 3 for Ag(I) ion and pH < 0 for Pd(II) desorption. Desorption time of Ag(I) and Pd(II) were shown in Fig. 5c. The desorption efficiency was reached 97.24 ± 0.21% after 120 min in Ag(I) adsorbed ATU-PEGDA hydrogels immersed in 1 M TU in 10^–3^ M HNO_3_ solution while the desorption efficiency reached 94.67 ± 1.52% after 120 min in Pd(II) adsorbed ATU-PEGDA hydrogels immersed in 1 M TU in 4 M HCl solution. The possible mechanism of Ag(I) and Pd(II) desorption might be considered as the competition of chelation and electrostatic force. In the cased on silver, ATU-PEGDA performed lower adsorption capacity when pH is 3. The main reason is that the protonation of amine group of ATU resulting in the generation of repulsive force between protonated ATU and silver ion. However, the TU molecules in elution can form the chelating complexes, [Ag(TU_3_)^+^] performing high stabilities. Therefore, the adsorbed silver ion would be released when the elution, 0.5 M TU in 10^–3^ M HNO_3_ elution. Similar desorption mechanism also be observed in Pd(II) desorption. The minimum adsorption capacity occurs at pH 1, which presents the competition adsorption of excessive chloride toward (PdCl_4_)^2−^. In addition, the TU molecules would form high stability Pd-chelated complexes, [Pd(Tu_4_)]^2+^. Consequently, the adsorbed Pd(II) would be extracted by 1 M TU in 4 M HCl elution. To summary, the mechanism of desorption of Ag(I) and Pd(II) from gel is constructed by the competition between chelation ability and electrostatic force. While the bonding of chelated complexes, like [Ag(TU_3_)^+^]and (PdCl_4_)^2−^ is stronger than electrostatic force, the adsorbed would be extracted from ATU-PEGDA gel adsorbents.

Under normal circumstances, the adsorption efficiency of an adsorbent is reduced in a repeated usage due to the damage of the material structure or the deterioration of the functional groups^25,33^. However, a salient observation was obtained in this regeneration experiment that the adsorption efficiency of ATU-PEGDA adsorbents was increased after regeneration and can be reused more than 10 times while still maintaining high adsorption efficiency. Effect of regeneration cycles on adsorption efficiency of the adsorbent on Ag(I) and Pd(II) were shown in Fig. 5d. After completing the first regeneration cycle for Ag(I) and Pd(II) loaded adsorbents, the adsorption efficiency was increased by 31.83 ± 3.08% and 75.39 ± 9.12% of pristine adsorbents, respectively. These values were compared between the first and second regeneration cycles and it was observed that the adsorption efficiency was increased till the 10th regeneration cycle. When compare the adsorption efficiencies of Ag(I) and Pd(II) loaded adsorbents between first and 10th regeneration cycles, it was increased by 33.83 ± 3.08% and 72.62 ± 14.95%, respectively.

**Figure 6:**
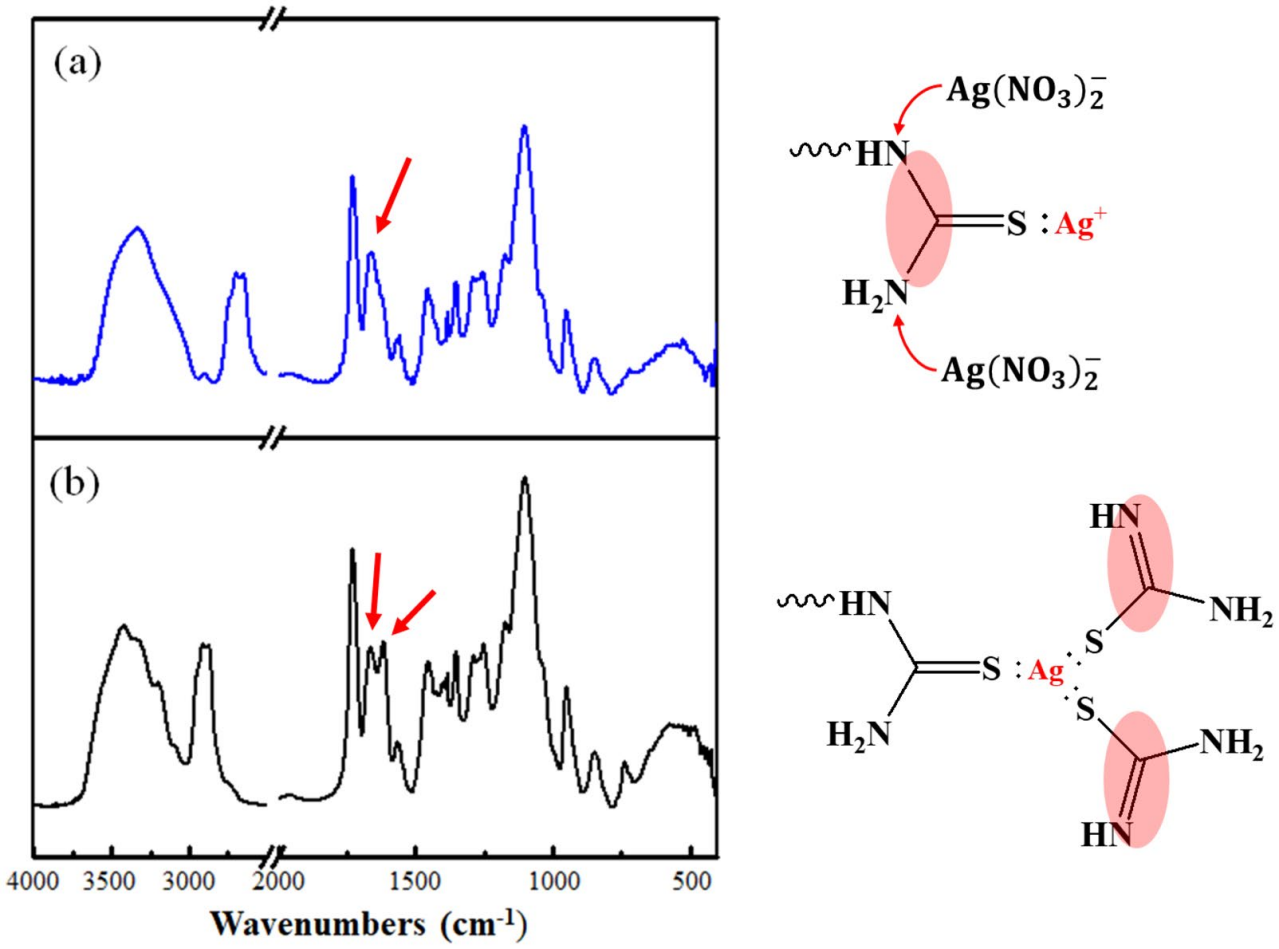
FTIR spectrum of (**a**) ATU-PEGDA. (**b**) Regenerated ATU-PEGDA.

**Figure 7:**
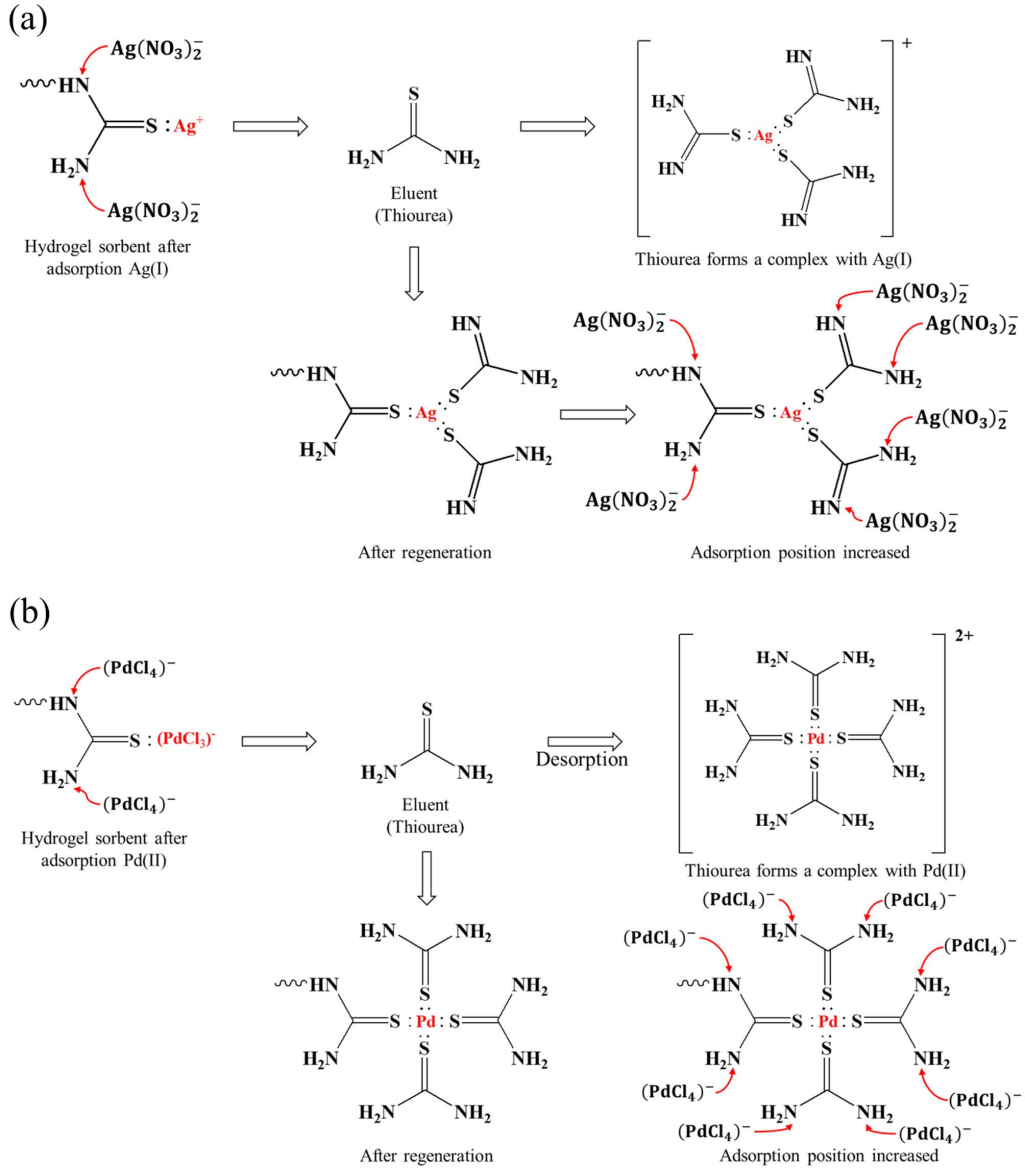
Possible adsorption mechanism of regenerated ATU-PEGDA for (**a**) silver ion; (**b**) palladium ion.

According to the Hard-Soft Acid–Base (HSAB theory by Pearson) theory; the soft metal ion Ag(I) has low effective ionic radii of 2.5 Å in an aqueous medium hence it has low ionic and high chelating interactions^3,5,7^. Therefore, chelating resins with functional groups containing sulfur and nitrogen as donor atoms chelate with Ag(I) ions while the affinity of soft metal ions to soft bases as S > N^4,5^. In the desorption reaction, thiourea in the desorption solution plays an important role. As shown in Fig. 6, thiourea with tautomers forms a stable complex with three thiourea molecules and one Ag^+^ called as the trithiourea silver(I) ion {Ag[SC(NH_2_)_2_]_3_^+^}/[Ag(TU_3_)^+^] and the double bond of the two thiourea molecules provided by the desorbent solution will be transferred to the nitrogen group^34^. This was proved by FT-IR analysis that a tensile vibration band was obtained around 1616 cm^−1^ as displayed in Fig. 6 revealing C=N which proved the double bond of thiourea molecule has transferred into the nitrogen group. Due to the desorption experimental results, about 1.6% of Ag^+^ cannot be desorbed as a result of the formation of [Ag(TU_3_)^+^] complex, which constructed additional TU branches in adsorbents. Consequently, regenerated ATU-PEGDA would perform higher adsorption capacity than pristine adsorbents. The mechanism was arranged in Fig. 7. Furthermore, similar adsorption behavior can also observe in regenerated ATU-PEGDA toward Pd(II) adsorption. The stable complex, tetrathiourea Palladium(II) ion {Pd[SC(NH_2_)_2_]_4_^2+^}/[Pd(TU_4_)^2+^] was formed in ATU-PEGDA during regeneration process resulting in improving the adsorption efficiency of regenerated adsorbents for Pd(II) in further regeneration cycle^35^.

**Figure 8:**
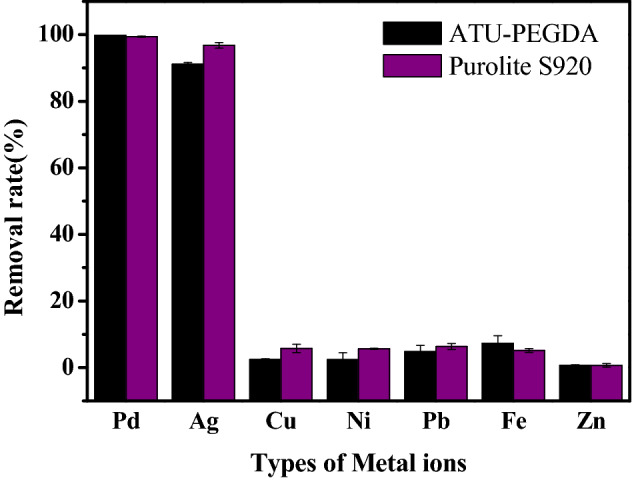
Removal efficiency of Ag(I) and Pd(II) in waste electrolyte by hydrogel adsorbent and Purolite S920 chelating resin at 25 °C (pH 2.43, W = 50 mg, V = 10 mg).

Figure 8 shows the comparison of adsorption capacities for Ag(I) and Pd(II) using ATU-PEGDA and commercialized chelating resin (Purolite S920). ATU-PEGDA performed similar adsorption behavior and adsorption selectivity as Purolite S920. Additionally, another observation was when ATU-PEGDA adsorbs Ag(I), the color of the adsorbent changed gradually from transparent to brown, and it turned into dark brown when it was close to the adsorption saturation. When adsorbing Pd(II), the color of the adsorbent gradually changed from transparent to dark red in equilibrium. This obviously color change might be due to the ligand-to-metal charge transfer (LMCT). The adsorption of Ag(I) and Pd(II) using ATU-PEGDA is contributed by formation [Ag(TU_3_)^+^] or [Pd(TU_4_)^2+^] complexes resulting in an electron migrating from thiourea-to-metal and metal-to-thiourea in addition to π–π* conjugation^30^. As a result, the ATU-PEGDA adsorbents exhibit color change after Ag(I) and Pd(II) adsorption shown in video S1.

## Materials and methods

### Materials

Polyethylene glycol diacrylate (PEGDA, MW: 700) and photoinitiator-2,2-dimtehoxy 2-phenylacetonphenone (C_16_H_16_O_3_, 96%) were purchased from Sigma-Aldrich. 1-vinyl-2-pyrrolidinone obtaining (C_6_H_9_NO, 96%) from ACROS was used as a solvent of photoinitiator. The modifier, allylthiourea (ATU, C_4_H_8_N_2_S, 98%) was obtained from Sigma-Aldrich. Metal ion, silver nitrate (AgNO_3_, 99%) and palladium nitrate (Pd(NO_3_)_2_, 99%) were purchased from ACROS. All above-mentioned reagents were used without any further purification.

### Synthesis of ATU-PEGDA adsorbents

Polyethylene glycol diacrylate (PEGDA) is the main structure of hydrogel adsorbent and the backbone structure is modified by allylthiourea. The final product is called as ATU-PEGDA hydrogel. The molar ratio between PEGDA to allylthiourea was controlled 1–2 into deionized water containing 10% of photoinitiator and then poured into an Ecoflex mold for solidification under 365 nm UV irradiation (100 W) in 3 min. All hydrogel adsorbents were cleaned with deionized (DI) water for 5 min and were dried at 65 °C. All adsorbents were synthesized in duplicate, and the size of the adsorbents was 2 mm × 2 mm × 2 mm.

### Characterization of hydrogel adsorbent and Ag(I)/Pd(II) analysis

Fourier transform infrared (FT-IR) spectroscopy was obtain by Thermo Nicolet Is5 to identify the structure of the synthesized adsorbent and adsorption mechanism. The wavenumber ranges from 400 to 4000 cm^−1^ were used, and the absorbance was recorded with 0.9 cm^−1^ resolution. Ag(I) and Pd(II) concentrations were determined by inductively coupled plasma atomic emission spectroscopy (ICP-OES, Perkin Elmer Optima 2000DV). The measured data were sampled and processed using the WinLab3ICP computer program.

### Ag(I) and Pd(II) adsorption and desorption experiments

To investigate the optimized conditions of adsorption capacity of ATU-PEGDA for Ag(I) and Pd(II) from an aqueous medium, experiments were carried out using 100 ppm initial concentration of Ag(I) and Pd(II). First, 10 mL of 100 ppm Ag(I) or Pd(II) and 50 mg of ATU-PEGDA were taken into a 20 mL vial having various pH from 1 to 6. The desired pH values were adjusted using 0.1 M HNO_3_ or 0.1 HCl and 0.1 M NaOH solutions. Then these vials were kept on a shaker for a certain time with the speed of 110 rpm at room temperature with time 40, 80, 120, 160, 200, 240, and 300 min.

Following the adsorption experiment (i.e., after 300 min of incubation), desorption experiments were conducted in 20 mL glass vials using a range of different extraction solutions, including various concentrations of thiourea (TU) dissolved in various concentrations of HNO_3_, H_2_SO_4,_ and HCl acids as desorption solutions. Briefly, the spent adsorbents in the vials were washed with DI water twice before adding 10 mL of extraction solution. The mixture was incubated at room temperature with 110 rpm for 24 h. The spent adsorbents after desorption were washed with DI water two times. Duplicate samples were used in each set of adsorption/desorption experiments and the process was repeated 10 times.

During the adsorption and desorption experiments, liquid samples were collected and analyzed using Inductively Coupled Plasma Optical Emission Spectrometry (ICP-OES). Ag(I) and Pd(II) adsorption capacities of ATU-PEGDA adsorbent were calculated according to the following equation (Eq. 8).

8 $${Q}_{e} =\frac{({C}_{o}-{C}_{e})V}{W}$$

where ${Q}_{e}$= Equilibrium adsorption capacity of adsorbent (mg g^−1^), ${C}_{o}$ = Initial concentration of precious metal in aqueous medium (mg L^−1^), ${C}_{e}$ = concentration of precious metal in aqueous medium after adsorption (mg L^−1^), V = volume of the metal ion solution (L), W = mass of the adsorbent (g)^20^.

### Adsorption kinetics and adsorption model

To evaluate the adsorption mechanism and to realize the potential rate-controlling steps of Ag(I) and Pd(II) ions on ATU-PEGDA, the pseudo-first-order and the pseudo-second-order kinetic models were employed. The linear forms of the pseudo-first-order and the pseudo-second-order could be expressed as Eqs. (9) and (10), respectively.

9 $$\mathrm{ln}({Q}_{e}-{Q}_{t})=\mathrm{ln}{Q}_{e}-{k}_{1}t$$

10 $$\frac{t}{{Q}_{t}}= \frac{1}{{k}_{2}{Q}_{e}^{2 }}+\frac{1}{{Q}_{e}}$$

where ${Q}_{e}$(mg g^−1^) was the equilibrium adsorption capacity of metal ion, ${Q}_{t}$ (mg g^−1^) was the amount of adsorbed metal ion at adsorption time t (min), ${k}_{1}$ (min^−1^) was rate constant of the pseudo-first-order and ${k}_{2}$ (g mg^−1^ min) was rate constant of the pseudo-second-order.

To interpret the adsorption experimental data, Freundlich and Langmuir models were applied. The Langmuir isotherm model and its linearized form can be expressed as Eqs. (11) and (12), respectively^3,20^. Separation factor (R_L_) was calculated as Eq. (13) using initial concentration and Langmuir constant,

11 $${\mathrm{Q}}_{\mathrm{e}}={\mathrm{Q}}_{\mathrm{max}}\frac{{\mathrm{K}}_{\mathrm{L}}{\mathrm{C}}_{\mathrm{e}}}{1+{\mathrm{K}}_{\mathrm{L}}{\mathrm{C}}_{\mathrm{e}}}$$

12 $$\frac{{\mathrm{C}}_{\mathrm{e}}}{{\mathrm{Q}}_{\mathrm{e}}}=\frac{1}{{\mathrm{Q}}_{\mathrm{max}}{\mathrm{K}}_{\mathrm{L}}}+\frac{{\mathrm{C}}_{\mathrm{e}}}{{\mathrm{Q}}_{\mathrm{max}}}$$

13 $${\mathrm{R}}_{\mathrm{L}}=\frac{1}{1+\left(1+{\mathrm{C}}_{0}{\mathrm{K}}_{\mathrm{L}}\right)}$$

where Q_max_ (mmol g^−1^) was the maximum adsorption capacity, C_e_ (mmol L^−1^) was the equilibrium concentration of Ag(I) and Pb(II), Q_e_ (mg g^−1^) was the equilibrium adsorption of Ag(I) and Pb(II) ions and K_L_ (g mg^−1^) was the Langmuir constant. The Freundlich isotherm model and its logarithmic form can be expressed as Eqs. (14) and (15), respectively^3,20^.

14 $${\mathrm{Q}}_{\mathrm{e}}={\mathrm{K}}_{\mathrm{F}}{{\mathrm{C}}_{\mathrm{e}}}^{\frac{1}{\mathrm{n}}}$$

15 $$\mathrm{ln}{Q}_{e}=\mathrm{ln}{K}_{F}+\frac{1}{n}\mathrm{ln}{C}_{e}$$

where K_F_ (mg g^−1^) was an indicative constant related to the adsorption capacity of the adsorbent and 1/n (0–1) was the adsorption intensity or surface heterogeneity of the adsorbent^20,36^.

### Selective adsorption and wastewater test

Selective adsorption of Ag(I) and Pd(II) from acid solutions with co-existing Cu^2+^, Ni^2+^, Pb^2+^, Zn^2+^ and Fe^3+^ with 100 ppm concentrations were investigated using ATU-PEGDA at pH 1. The volume of the two solutions was 10 mL and 5 mg of hydrogel was added to each. Then both solutions were shaken at 110 rpm for 24 h at room temperature. After that, the concentrations of Ag(I), Pd(II) and the coexistent metal ions were determined by ICP-OES. The adsorption capacities of each metal ion were calculated according to Eq. (8) and the adsorption selectivity was determined by Eq. (16)^36,37^.

16 $${\mathrm{Sel}}_{\mathrm{i}}=\mathrm{log}\frac{{\left({\mathrm{Q}}_{\mathrm{e}}/{\mathrm{C}}_{\mathrm{e}}\right)}_{\mathrm{i}}}{{\sum \left({\mathrm{Q}}_{\mathrm{e}}/{\mathrm{C}}_{\mathrm{e}}\right)}_{\mathrm{j}}}$$

where Q_e_ is the amount of metal adsorbed at equilibrium per unit weight of adsorbent (mmol g^−1^); C_e_ is the equilibrium concentration of metal ions in solution (mmol L^−1^), the index i pertains to Ag or Pd and index j refers to the remaining metals in the solution other than i (Cu^2+^, Ni^2+^, Pb^2+^, Zn^2+^, Fe^3+^).

The waste electrolytic sample was taken from the field and silver-palladium alloy ingot was obtained after pretreatment of electrolytic sample. After degrade the electrolyte; (excessive other metal impurities) saturated NaCl was added to the electrolyte to react with Ag(I) ions. Then the precipitated AgCl was separated from the electrolyte and the remaining metal concentrations of the electrolyte were determined by ICP-OES. Next, 10 mL of electrolytes were mixed with 50 mg of traditional macro porous thiourea chelating resin (brand: Model S920) and ATU-PEGDA hydrogels separately. After that, both samples were shaken with 110 rpm for 24 h at room temperature. Finally, the supernatant of both samples were analyzed using ICP-OES for determine the remaining metal ion concentrations.

## Conclusion

This is the first report to develop ATU-PEGDA hydrogel-based adsorbent for selectively adsorption toward Ag(I) and Pd(II) from aqueous medium. ATU-PEGDA reached adsorption equilibrium in 240 min and 300 min for Ag(I) and Pd(II), respectively. The optimized conditions for these precious metal ions were occurred at pH 1 and even lower acidity. Pseudo-first-order kinetic model and Langmuir adsorption isotherm can fully describe the adsorption behavior of ATU-PEGDA toward Ag(I) and Pd(II). Adsorption efficiencies of both metal ions were higher than 99.8% (< 1 ppm) and adsorption capacities of both metal ions were 83.33 mg Ag(I)/g adsorbents and 152.81 mg Pb(II)/g adsorbents. ATU-PEGDA adsorbents can sustainably capture Ag(I) and Pd(II) under several adsorption–desorption cycles without any damage. Furthermore, the regenerated ATU-PEGDA adsorbents exhibited superior to pristine adsorbents for capturing Ag(I) and Pd(II) because of the formation of thiourea-metal complexes. These complexes not only constructed additional adsorption sites in adsorbents but also generated ligand-to-metal charge transfer (LMCT) in coloration after adsorption. ATU-PEGDA adsorbents performed excellent adsorption behavior and selectivity of Ag(I) and Pd(II) occurring in extremely lower pH of the solution containing multiple metal ions. It is also expected that the ATU-PEGDA adsorbents can potentially apply in PCB wastewater for direct extraction and condensation of Ag(I) and Pd(II) without any pretreatments. Most commercialized thiourea-based resins are constructed by polystyrene. Therefore, it requires organic solvents, like dichloromethane (DCM) or dimethyl furan (DMF) to swell the polystyrene resins resulting in secondary pollution during functionalization. ATU-PEGDA hydrogel-based adsorbents are synthesized in aqueous environment indicating these adsorbents can be considered as eco-friendly products. The solidification of ATU-PEGDA is processed under UV irradiation in short time. Therefore, it has benefit to massive production of ATU-PEGDA integrating with automatic system, like roll-to-roll manufacturing. In addition, PEGDA composes highly hydrophilic structure, like ether, in order to easily uptake and recycle previous metal ion from wastewater. Furthermore, this hydrophilicity also effective improve the regeneration efficiency. The excellent renewability of green adsorbents, ATU-PEGDA not only would effectively reduce the cost of operation but also would minimize the secondary pollution during manufacturing. Based on these advantages and excellent properties, ATU-PEGDA adsorbents can be further developed for continuous treatment of precious metal ion recycling using packed and/or fluidized bed reactors.

## Supplementary Information

Supplementary Information 1.

Supplementary Video 1.
