# Supplementary material for: Novel reusable hydrogel adsorbents for precious metal recycle
Source: Sci Rep. 2021 Oct 1;11:19577. doi: 10.1038/s41598-021-99021-5 (PMC8486833; doi:10.1038/s41598-021-99021-5)
Supplement: Supplementary file 1 — Supplementary Information 1. [file 41598_2021_99021_MOESM1_ESM.docx]

**Supplementary data**

**Novel Reusable Hydrogel Adsorbents for Precious Metal Recycle**

by

Thakshila Nadeeshani Dharmapriya^1^, Ding-Yang Lee^1^, and Po-Jung Huang^1*^

^1^Institute of Environmental Engineering, National Sun Yat-sen University, Kaohsiung, 80432

* corresponding author, email: [pjhuang@mail.nsysu.edu.tw](mailto:pjhuang@mail.nsysu.edu.tw)

**Table S1.** Adsorption kinetic analytic data for ATU-PEGDA toward Ag(I) and Pg(II)

| Metal ion | Q_e_ (mg/g) Experimental | Pseudo-first-order kinetic model | | |
| --- | --- | --- | --- | --- |
|  |  | k_1_ (minute^-1^) | Q_e_ (mg/g) | R^2^ |
| Ag^+^ | 64.63 | 0.0301 | 67.89 | 0.99672 |
| Pd^2+^ | 111.99 | 0.0236 | 105.99 | 0.99072 |
| Metal ion | Q_e_ (mg/g) Experimental | Pseudo-second-order kinetic model | | |
|  |  | k_2_ (g/mg min) | Q_e_ (mg/g) | R^2^ |
| Ag^+^ | 64.63 | 7.56$\times$ 10^-5^ | 101.01 | 0.97893 |
| Pd^2+^ | 111.99 | 5.90$\times$ 10^-5^ | 153.85 | 0.97660 |

**Table S2**. Adsorption isotherm analytic data for ATU-PEGDA toward Ag(I) and Pd(II)

| Temperature（℃） | Metal ions | Langmuir isotherm | | | | | |
| --- | --- | --- | --- | --- | --- | --- | --- |
|  |  | Q_max_ (mg/g) | K_L_ (L/mg) | | R_L_ | R^2^ | |
| 25 | Ag^+^ | 83.33 | 0.1214 | | 0.0381 | 0.99468 | |
| 25 | Pd^2+^ | 152.81 | 0.1343 | | 0.0349 | 0.99870 | |
| Temperature（℃） | Metal ions | Freundlich isotherm | | | | | |
|  |  | n | | K_F_（L/mg） | | | R^2^ |
| 25 | Ag^+^ | 4.06240 | | 0.49918 | | | 0.98475 |
| 25 | Pd^2+^ | 5.47196 | | 0.62436 | | | 0.95048 |

**Table S3**. Adsorption selectivity data

| Metal ions | Q_e_ (mg/g) | C_e_ (mg/L) | Selective coefficient |
| --- | --- | --- | --- |
| Pd^2+^ | 118.12 | 47.06 | 1.31 |
| Ag^+^ | 61.61 | 65.38 | 0.89 |
| Cu^2+^ | 2.87 | 106.83 | -1.59 |
| Ni^2+^ | 2.09 | 105.73 | -1.72 |
| Fe^3+^ | 2.01 | 99.77 | -1.71 |
| Pb^2+^ | 2.48 | 122.13 | -1.71 |
| Zn^2+^ | 3.38 | 98.19 | -1.48 |
